# Frequent *ATRX*, *CIC*, *FUBP1* and *IDH1* mutations refine the classification of malignant gliomas

**DOI:** 10.18632/oncotarget.588

**Published:** 2012-08-03

**Authors:** Yuchen Jiao, Patrick J. Killela, Zachary J. Reitman, B. Ahmed Rasheed, Christopher M. Heaphy, Roeland F. de Wilde, Fausto J. Rodriguez, Sergio Rosemberg, Sueli Mieko Oba-Shinjo, Suely Kazue Nagahashi Marie, Chetan Bettegowda, Nishant Agrawal, Eric Lipp, Christopher J. Pirozzi, Giselle Y. Lopez, Yiping He, Henry S. Friedman, Allan H. Friedman, Gregory J. Riggins, Matthias Holdhoff, Peter Burger, Roger E. McLendon, Darell D. Bigner, Bert Vogelstein, Alan K. Meeker, Kenneth W. Kinzler, Nickolas Papadopoulos, Luis A. Diaz, Hai Yan

**Affiliations:** ^1^ Ludwig Center for Cancer Genetics and Howard Hughes Medical Institutions, The Johns Hopkins Kimmel Cancer Center, the Department of Oncology, the Department of Pathology, the Department of Neurosurgery, the Johns Hopkins Medical Institutions, Baltimore, Maryland, USA; ^2^ The Preston Robert Tisch Brain Tumor Center at Duke, The Pediatric Brain Tumor Foundation Institute, the Department of Pathology, the Department of Surgery, Duke University Medical Center, Durham, North Carolina, USA; ^3^ The Department of Pathology, the Department of Neurology, School of Medicine, University of Sao Paulo, Sao Paulo, Sao Paulo, Brazil; ^4^ The Swim Across America Laboratory at Johns Hopkins, The Johns Hopkins Medical Institutions, Baltimore, Maryland, USA

**Keywords:** ALT, IDH1, IDH2, Mixed Gliomas

## Abstract

Mutations in the critical chromatin modifier *ATRX* and mutations in *CIC* and *FUBP1*, which are potent regulators of cell growth, have been discovered in specific subtypes of gliomas, the most common type of primary malignant brain tumors. However, the frequency of these mutations in many subtypes of gliomas, and their association with clinical features of the patients, is poorly understood. Here we analyzed these loci in 363 brain tumors. *ATRX* is frequently mutated in grade II-III astrocytomas (71%), oligoastrocytomas (68%), and secondary glioblastomas (57%), and *ATRX* mutations are associated with *IDH1* mutations and with an alternative lengthening of telomeres phenotype. *CIC* and *FUBP1* mutations occurred frequently in oligodendrogliomas (46% and 24%, respectively) but rarely in astrocytomas or oligoastrocytomas (<10%). This analysis allowed us to define two highly recurrent genetic signatures in gliomas: *IDH1/ATRX* (I-A) and *IDH1/CIC/FUBP1* (I-CF). Patients with I-CF gliomas had a significantly longer median overall survival (96 months) than patients with I-A gliomas (51 months) and patients with gliomas that did not harbor either signature (13 months). The genetic signatures distinguished clinically distinct groups of oligoastrocytoma patients, which usually present a diagnostic challenge, and were associated with differences in clinical outcome even among individual tumor types. In addition to providing new clues about the genetic alterations underlying gliomas, the results have immediate clinical implications, providing a tripartite genetic signature that can serve as a useful adjunct to conventional glioma classification that may aid in prognosis, treatment selection, and therapeutic trial design.

## INTRODUCTION

Gliomas are the most common primary tumors of the central nervous system. They are classified as grade I to grade IV on the basis of histological criteria established by the World Health Organization (WHO) [1]. Besides grading, gliomas are further classified into subtypes of which the most common include astrocytomas and oligodendrogliomas. Grade I gliomas are usually benign and operable, and rarely progress to higher-grade gliomas. Grade II astrocytomas and oligodendrogliomas can progress to grade III anaplastic astrocytomas and anaplastic oligodendrogliomas, respectively. Grade III gliomas are generally incurable and have a median survival of 3-10 years. Glioblastomas (GBMs) are grade IV astrocytomas including mainly “primary GBMs” (95%) that arise *de novo*, and “secondary GBMs” that have progressed from clinically discernible grade II or III astrocytomas. Grade II-III oligoastrocytomas are a subset of gliomas that present with an appearance of mixed glial cell origin, astrocytoma and oligodendroglioma [1]. These “mixed gliomas” are particularly difficult to diagnose and their interpretation is often subjective and varies between different pathologists [1-3].

Recent research has begun to characterize the molecular landscape of gliomas to better understand their molecular pathogenesis and to aid in their classification for clinical management. High-throughput molecular studies by The Cancer Genome Atlas and other groups have focused on the more common and lethal grade IV GBMs and identified molecular subgroups of those tumors [4-8]. Major findings include frequent activating mutations in isocitrate dehydrogenase genes, *IDH1* and *IDH2* (“IDH mutation” refers to a mutation in either gene), in secondary GBMs (>50%) and rarely in primary GBMs (<5%) [5, 9-12]. Additionally, inactivating alterations in *Alpha Thalassemia/Mental Retardation Syndrome X-linked* (*ATRX*) were recently identified in 7% of adult GBMs and in 14-31% of pediatric GBMs [13, 14]. ATRX is critical for normal telomere homeostasis by regulating incorporation of histone variant H3.3 into telomeric chromatin [15-17], and *ATRX* alterations are associated with an alternative lengthening of telomeres (ALT) phenotype among GBMs [13, 14]. Recent GBM exome sequencing studies also confirmed frequent mutations in the tumor suppressor gene *TP53* among secondary GBMs (65%), and less frequently in primary GBMs (28%) [5, 6, 14, 18].

The molecular properties of grade II-III gliomas are less well-characterized than grade IV GBMs. Lower-grade gliomas share molecular features with secondary GBMs and with a subset of primary GBMs, including frequent *IDH* mutations (>70%) [10, 12, 19], a proneural gene expression signature [6, 7, 20], and a glioma CpG island hypermethylator phenotype (G-CIMP) [8]. Grade II-III astrocytomas frequently harbor *TP53* mutations, which are uncommon in oligodendrogliomas [3, 10]. ALT occurs frequently in grade II-III astrocytomas (63%) but less commonly in oligodendrogliomas (20%) [21-24]. However, whether *ATRX* mutations occur in adult grade II-III astrocytomas, and whether they may associate with ALT in those tumors, remains unknown.

Co-deletion of chromosomal arms 1p and 19q is a well-known prognostic marker found frequently (>60%) in grade II-III oligodendrogliomas [25-27]. Recent exomic sequencing of oligodendrogliomas revealed inactivating mutations in two tumor suppressor genes: *homolog of Drosophila capicua (CIC)* and *far-upstream binding protein 1 (FUBP1)* in 53% and 15% of oligodendrogliomas, respectively [28-30]. Since *CIC* is located on chromosomal arm 19q and *FUBP1* is located on chromosomal arm 1p, 1p/19q loss is thought to be a mechanism to inactivate *CIC* and *FUBP1* [28]. Among grade II-III gliomas, *CIC* and *FUBP1* mutational status has only been investigated in oligodendrogliomas, oligoastrocytomas and preselected astrocytomas with 19q loss [30].

Given the difficulty in classifying gliomas, in particular grade II-III gliomas, we sought to characterize the landscape of genetic alterations in numerous common subtypes of brain tumors. We focused on both alterations that have been discovered recently in at least one glioma subtype by high-throughput molecular analyses (*ATRX*, *CIC*, *FUBP1*) and on classic alterations (*IDH*, *TP53*, 1p/19q) that could be routinely detected in a molecular genetics laboratory. This analysis uncovered frequent alterations in *ATRX*, *CIC*, and *FUBP1* in new glioma subtypes and identified an association between *ATRX* alterations and ALT in grade II-III gliomas. The mutational pattern identified here also revealed mutually exclusive, highly recurrent, multi-gene mutational signatures that correlated well with the clinical features of the patients.

## RESULTS

### ATRX inactivation is linked to mutations in TP53 and IDH, and to altered telomeres

**Figure 1:**
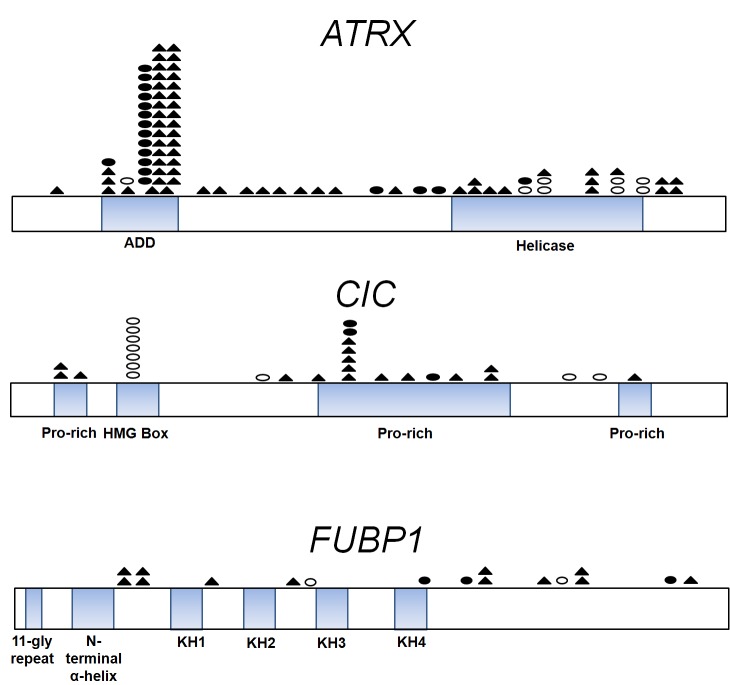
Distribution of ATRX, CIC, and FUBP1 mutations in gliomas Schematics of the coding sequences of ATRX, CIC, and FUBP1 are shown. Mutations were predominantly truncating mutations such as frameshifts (shaded triangles) and nonsense mutations (shaded circles). Empty circles correspond to missense mutations. ATRX contains a DNA-binding ATRX-DNMT3-DNMT3L (ADD) domain and an ATPase Helicase Domain. ATRX alterations occurred in 20% of pediatric GBMs (n=25) and all of those mutations clustered in the Helicase domain of ATRX as described previously [14]. ATRX mutations in adult gliomas instead distributed evenly. CIC contains four highly conserved domains, the HMG (high mobility group) box responsible for DNA binding and proline-rich (pro-rich) domains. Among CIC mutations (n=29), a predilection for missense mutations occurred in the HMG box domain (24%). Mutations in FUBP1 (n=16) occurred 3' of the N-terminal α-helix domain, which controls binding of FUBP1 to the FBP-interacting repressor (FIR). Gene representations are to scale.

*ATRX* alterations were found in 93 out of 363 brain tumors, in ten tumor subtypes (Table 1). *ATRX* alterations occurred frequently in grade II astrocytomas (67%, n=15), grade III astrocytomas (73%, n=44), secondary GBMs (57%, n=14), and in tumors of mixed astrocytic and oligodendrocytic lineage (68%, n=40). In contrast, *ATRX* alterations were rare in primary GBMs (4%, n=94) and uncommon in pediatric GBMs (20%, n=25) and pure oligodendroglial tumors (14%, n=50). Surprisingly, while all of the *ATRX* mutations in pediatric GBMs were found near the carboxy-terminal Helicase domain as observed previously [14, 31], mutations in adult gliomas instead distributed evenly (Figure 1 and Supplementary Table 1).

**Table 1:** Frequency of mutations in 13 tumor types

					IDH 1/2		ATRX		TP53		CIC		FUBP1	
Tumor Classification	No. of tumors analyzed	Mean age (yrs.)	Median age (yrs.)	Male Sex (%)	No.	%	No.	%	No.	%	No.	%	No.	%
Astrocytic tumors	203	37	26	54%	71	35%	59	29%	91	45%	1	0.50%	2	1%
Pilocytic Astrocytoma (grade I)	9	7	4	56%	0	0%	0	0%	NA	NA	0	0%	0	0%
Pleomorphic xanthoastrocytoma (grade II)	2	11	11	0%	0	0%	0	0%	2	100%	0	0%	0	0%
Astrocytoma (grade II)	15	25	30	60%	10	67%	10	67%	9	60%	0	0%	0	0%
Anaplastic Astrocytoma (grade III)	44	36	35	61%	37	84%	32	73%	36	82%	0	0%	0	0%
Secondary Adult glioblastoma (grade IV)	14	37	36	64%	8	57%	8	57%	11	79%	0	0%	0	0%
Adult glioblastoma (grade IV)	94	56	57	60%	13	14%	4	4%	22	23%	1	1%	2	2%
Pediatric glioblastoma (grade IV)	25	12	12	79%	3	12%	5	20%	11	44%	0	0%	0	0%
														
Oligodendroglial	50	40	41	55%	46	92%	7	14%	7	14%	23	46%	12	24%
Oligodendroglioma (grade II)	21	36	35	48%	18	86%	5	24%	5	24%	8	38%	3	14%
Anaplastic Oligodendroglioma (grade III)	29	43	46	62%	28	96%	2	7%	2	7%	15	52%	9	31%
														
Oligoastrocytic (Mixed)	40	41	38	58%	39	98%	27	68%	27	68%	3	8%	2	5%
Oligoastrocytoma (grade II)	18	42	39	61%	18	100%	10	56%	11	61%	1	6%	1	6%
Anaplastic Oligoastrocytoma (grade III)	22	40	37	55%	21	95%	17	77%	16	73%	2	9%	1	5%
														
Ependymoma (grade II)	5	NA	NA	NA	0	0%	0	0%	NA	NA	0	0%	0	0%
														
Medulloblastoma (grade IV)	65	9	7	54%	0	0%	1	2%	1	NA	2	3%	0	0%

**Figure 2:**
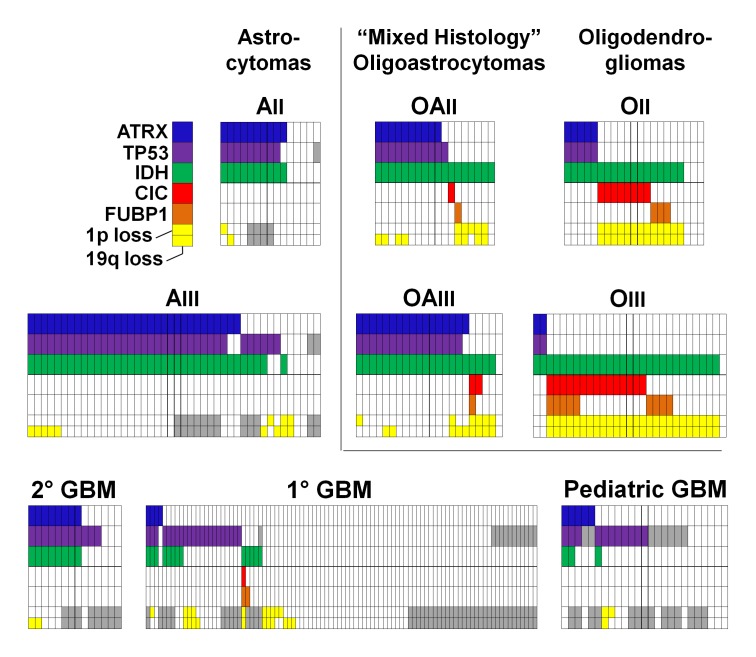
Distribution of ATRX, TP53, IDH, CIC, and FUBP1 mutations, and of chromosomes 1p and 19q loss, in grade II-IV gliomas Data are from 15 grade II astrocytomas (AII), 44 grade III astrocytomas (AIII), 21 grade II oligodendrogliomas (OII), 29 grade III oligodendrogliomas (OIII), 18 grade II oligoastrocytomas (OAII), 22 grade III oligoastrocytomas (OAIII), 14 secondary GBMs (2° GBM), 94 primary GBMs (1° GBM), and 25 pediatric GBMs. chromosome 1p status is indicated in the top of the lower row, and chromosome19q status is indicated in the bottom of this cell, with yellow coloring indicating loss of the indicated chromosomal arm. Gray cells denote analyses which were not informative or for which additional genetic material was not available for analysis.

**Figure 3:**
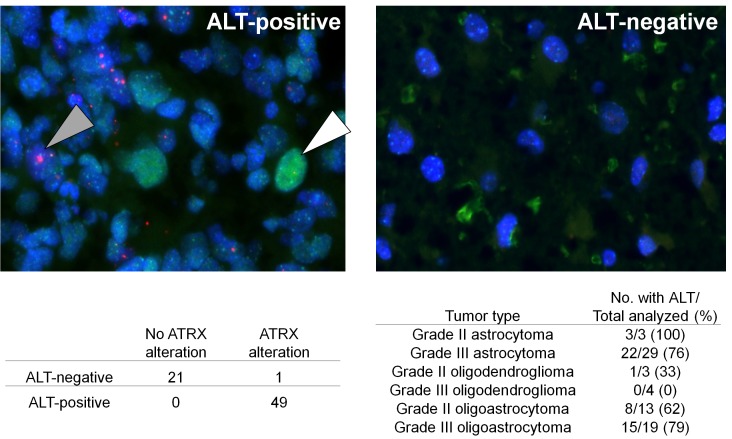
ALT associates with the ATRX alterations and the I-A signature (A) representative grade III astrocytoma section that was positive for ALT is shown on the left. A nucleus demonstrating large, ultra-bright telomeres (pink) is indicated with the shaded arrow. A p53 positive nucleus (green), which reflects p53 dysfunction, is indicated with the empty arrow. A representative image of a grade III astrocytoma section that was negative for both ALT and for p53 nuclear staining is shown on the right. The number of tumors with ALT and/or ATRX alterations is shown, along with the percentage of each tumor type with ALT. All tumors with ATRX alterations were I-A, that is, they also had an *IDH* mutation (n=50); the remaining tumors were *IDH* wild-type and did not have an ATRX alteration (n=21).

The association between *ATRX* alterations and other frequent molecular alterations was analyzed among the grade II-IV gliomas (Figure 2 and Supplementary Fig. 1). *IDH* mutations were observed in 87 of 88 adult gliomas with an *ATRX* alteration (99%). We termed tumors with this genetic signature “I-A gliomas”, denoting that these tumors had alterations in either *IDH1* or *IDH2* and in *ATRX*. Most I-A adult gliomas also had mutations in *TP53* (94%, n=87), and the majority of adult gliomas with *TP53* mutations had the I-A signature (72%, n=113). The I-A signature was tightly associated with ALT among grade II-III gliomas (n=71): 100% (49/49) of gliomas with ALT were I-A, and 98% (49/50) of gliomas with I-A were ALT positive (*P* <0.001, Figure 3). *ATRX* alterations and 1p/19q loss rarely co-occurred (only one case, *P* <0.001).

### CIC and FUBP1 mutations are linked to IDH mutation and 1p/19q loss

Sequencing analysis of *CIC* and *FUBP1* genes in the same brain tumors revealed 29 and 16 somatic mutations, respectively (Figure 1 and Supplementary Table 1). The highest frequency of *CIC* and *FUBP1* mutations occurred in grade II (38% and 14%, n=21) and grade III oligodendrogliomas (52% and 31%, n=29). In contrast, *CIC* and *FUBP1* mutations were rare in primary GBMs (1% and 2%, n=94) and completely absent (0%, n=59) in grade II-III pure astrocytic tumors. Seven tumors had concurrent *CIC* and *FUBP1* mutations. Every glioma with a *CIC* or an *FUBP1* mutation exhibited an *IDH* gene mutation (Figure 2 and Supplementary Fig. 1). Almost every glioma with a *CIC* or an *FUBP1* mutation exhibited loss of both chromosomes 1p and 19q (98%, n=36). Therefore, we termed tumors that had mutations in *IDH1* or *IDH2* combined with either 1p/19q loss, *CIC* mutation, or *FUBP1* mutations as “I-CF gliomas.” *CIC* and *FUBP1* mutations were mutually exclusive with *ATRX* or *TP53* mutations (*P* <0.001 for all four comparisons).

For the purposes of this study, other adult gliomas that did not fall into the I-A or I-CF signatures were categorized as I-X gliomas (i.e. not I-A or I-CF). Nearly half of adult gliomas included in this study (47%, n=257) were classified as I-X gliomas. Seventy-four percent of the I-X gliomas were diagnosed as grade IV primary GBMs and 95% of primary GBMs were classified as I-X gliomas. Most, but not all I-X gliomas (87%) were *IDH* wild type. Mutant *TP53* was found in 26% of these tumors, and most of the I-X gliomas with *IDH* mutations (69%) also had *TP53* mutations. Seven percent of adult gliomas were *ATRX* mutant or showed loss of 1p and 19q and were considered I-X gliomas because they lacked *IDH* mutations.

### Pathologic and clinical characteristics of I-A, I-CF and I-X gliomas

On the basis of the mutational patterns described above, gliomas could be classified as either I-A, I-CF, or I-X. We sought to determine whether these glioma genetic signatures were associated with any differences between the clinical characteristics of the patients who bore the tumors. Survival, age at diagnosis, and histopathological diagnosis data were collected for 199 grade II-IV adult glioma patients, including a cohort of glioma patients from the United States (n=156 patients) and a cohort of glioma patients from Brazil (n=43 patients). Age, median survival, and histopathological diagnosis of patients with I-A, I-CF, and I-X tumors were similar in the two cohorts (Supplementary Table 2). Distribution of genetic signatures among the combined cohort of 199 patients is shown in Table 2 and was as follows: gliomas classified as astrocytic were primarily I-A (32%) or I-X (66%) and rarely carried the I-CF (2%) genetic signature. Oligodendroglial tumors were primarily I-CF (77%) but a significant proportion harbored the I-A (16%) or I-X (7%) genetic signatures.

**Table 2:** Characteristics of patients with I-A, I-CF, and I-X genetic signatures

	I-A	I-CF	I-X	p Value*
Age (mean ± S.D., years)	34 ± 7	44 ± 11	54 ± 15	<0.001
(median, years)	33	46	56	
Gender (% male)	56	54	65	0.33
				
Survival (median, months)	51	96	13	<0.001
				
All Cases (n=199)	59 (29.6)	39 (19.6)	101 (50.8)	
				
Astrocytomas (n=148)	48 (32.4)	3 (2.0)	97 (65.6)	
				
Oligodendrogliomas (n=44)	7 (15.9)	34 (77.3)	3 (6.8)	
				
Mixed Histology (n=7)	4 (57.1)	2 (28.6)	1 (14.3)	
				
Grade II (n=31)	15 (48.4)	12 (38.7)	4 (12.9)	
				
Grade III (n=69)	34 (49.3)	25 (36.2)	10 (14.5)	
				
Grade IV (n=99)	10 (10.1)	2 (2.0)	87 (87.9)	
				
Secondary GBM (n=12)	7 (58.3)	0 (0.0)	5 (41.7)	
				
IDH Mutant Tumors (n=111)	59 (53.2)	39 (35.1)	13 (11.7)	
				

The 199 patients analyzed above are the subset of all adult (>20 yr) grade II-IV glioma patients who had age and survival information

* One-way ANOVA for difference in mean age distribution, Chi-squared test for difference in gender proportions, and log-rank test for difference in survival

Age at diagnosis and survival parameters were first analyzed among the group of 199 grade II-IV glioma patients, and then among subgroups of patients with specific tumor types. At the time of diagnosis, patients with I-A gliomas were younger (mean age of 34) than patients with I-CF tumors (mean age of 44, *P* <0.001) and patients with I-X tumors (mean age of 54, *P* <0.001). This stratification in age was independent of tumor grade, as patients with I-A tumors were younger than patients with the other two signatures among the group of grade III patients (34, 46, and 46 years old, *P* <0.001) and the group of grade IV patients (29, 53, and 55 years old, *P* <0.001).

**Figure 4:**
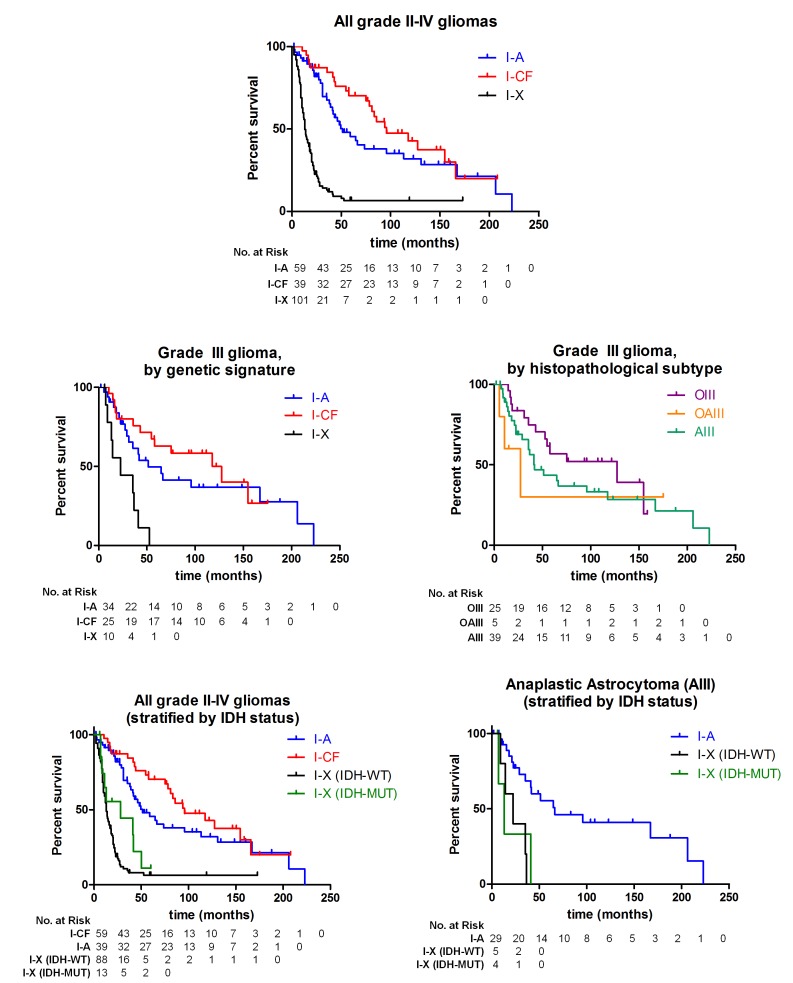
Survival of glioma patients with I-A, I-CF, and I-X genetic signatures Kaplan-Meier estimates of survival are shown for adult glioma patients, with the number of patients at risk at each time point for each group shown below the respective plot. Among all adult patients with grade II-IV gliomas (n=199), those with I-A tumors and with I-CF tumors survived significantly longer (median 51 and 96 months) than patients with I-X tumors (13 months; *P* <0.001 for both comparisons). Among grade III glioma patients with tumors of all histologies (n=69), there was a significant difference in survival between groups of patients with I-A or I-CF tumors and patients with I-X tumors (I-A and I-X, *P* = 0.002; I-CF and I-X, *P* <0.001). There was not a significant difference in survival when the same grade III glioma patients were stratified by the histopathological diagnosis of their tumors (*P* = 0.37). When all 199 grade II-IV gliomas were additionally stratified by *IDH* status, patients with I-A tumors and patients with I-CF tumors still survived significantly longer (median 51 and 96 months) than patients with *IDH*-mutated I-X tumors, and also longer than patients with *IDH*-WT I-X tumors (median 28 and 13 months, respectively; *P* <0.001 for all four comparisons). When grade III astrocytoma patients (n=39) were stratified by genetic signature and *IDH* status of their tumors, patients with I-A tumors survived longer (66 months) than grade III astrocytoma patients with either *IDH*-mutated I-X tumors (22 months, *P* = 0.005) and patients with *IDH*-WT I-X tumors (13 months, *P* = 0.002).

Among all grade II-IV glioma patients, those whose tumors bore the I-A and I-CF signatures survived longer than those with I-X tumors (median 51, 96, and 13 months, *P* <0.001) (Figure 4). Even when stratified by grade, patients whose tumors bore the I-A and I-CF signature survived longer than patients with the I-X signature among the subgroup of grade III gliomas (median 51, 127, and 22 months, *P* <0.001, Figure 4). In contrast, there was not a significant difference in survival between groups of grade III gliomas that were stratified by standard histopathological diagnostic criteria (*P* = 0.37, log-rank test, Figure 4). I-A patients also survived longer than I-X patients among GBMs (median 31 vs. 13 months, *P* = 0.016,) and among all astrocytomas (grade II-IV, median 51 vs. 13 months, *P* <0.001) (Supplementary Fig. 2). Significant differences in median survival between I-A, I-CF, and I-X signatures were not observed among the grade II glioma patients or grade II-III oligodendrogliomas, or between *CIC* and *FUBP1* mutated patients (Supplementary Fig. 2).

*IDH* mutations are associated with improved survival in gliomas [10, 11, 32]. Among all gliomas with *IDH* mutations, patients with *IDH* mutations in the I-X group had a shorter median survival (28 months) than patients with I-A tumors (51 months, *P* = 0.007) or I-CF tumors (96 months, *P* <0.001) (Figure 4). In the grade III astrocytoma group, patients with I-A tumors survived much longer (66 months) than patients with *IDH*-mutated I-X tumors (13 months, *P* = 0.002) and *IDH* wild-type I-X tumors (22 months, *P* = 0.005) (Figure 4). These results suggest that the I-A and I-CF signatures may provide more prognostic information than *IDH* mutational status alone.

### I-A and I-CF signatures can discriminate gliomas with mixed histopathology

40 gliomas with mixed oligodendrocytic and astrocytic histopathology were examined to determine their I-A, I-CF, and I-X status (Supplementary Table 3). 33 of these cases showed incomplete loss at loci on 1p36, 1p32, and 19q in prior clinical FISH testing and exhibited light microscopic appearances that were considered to be equivocal between oligodendroglioma or astrocytoma based on nuclear morphology and cytologic appearance. Greater than half of all mixed histology gliomas were I-A (65%), although I-CF (23%) and I-X (10%) genetic signatures were also found in this subgroup. In one case, the tumor had a “mixed” genetic signature: one grade III oligoastrocytoma demonstrated both an *ATRX* alteration and 1p/19q loss. When grouped by genetic signature, the age of oligoastrocytoma patients showed the same pattern observed for other glioma types: mean age at diagnosis was 35 for I-A patients, 49 for I-CF patients, and 59 for I-X patients (*P* = 0.001). Survival statistics were not tabulated because this cohort was only recently collected and median follow-up was insufficient.

## DISCUSSION

**Figure 5:**
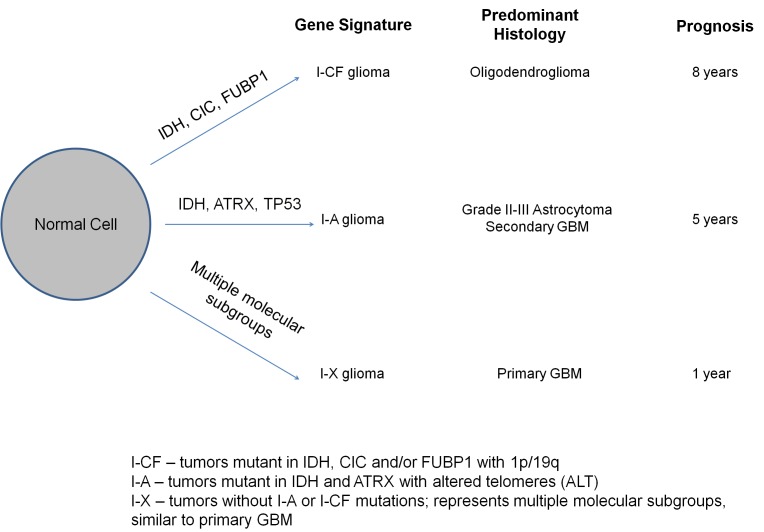
Model for molecular classification of gliomas Alterations in IDH and ATRX comprise the I-A signature, and alterations in IDH as well as one or more of CIC, FUBP1, or combined 1p/19q loss comprise the I-CF signature. I-X gliomas do not have either signature and likely represent multiple molecular disease subgroups. The I-A and I-CF signatures arise early in the pathogenesis of a glioma from a normal precursor cell. I-CF gliomas are typically grade II-III oligodendrogliomas and patients with these tumors survive for about 8 years. I-A gliomas are typically grade II-III astrocytomas or grade IV secondary GBMs and patients with these tumors survive for about 5 years. I-X gliomas are typically grade IV primary GBMs and patients with these tumors survive for about 1 year.

The mutational analysis of 363 brain tumors reported here represents the largest effort to date to define the distribution of *ATRX*, *CIC*, and *FUBP1* mutations in gliomas. This analysis revealed for the first time to our knowledge that *ATRX* mutations are frequent among grade II-III adult astrocytic tumors and are tightly associated with an ALT phenotype. The association between *ATRX* mutations with *IDH* mutations, and the association between *CIC/FUBP1* mutations and *IDH* mutations as well as 1p/19q loss defined I-A and I-CF genetic signatures. These signatures are especially common among the grade II-III gliomas and remarkably homogeneous in terms of genetic alterations and clinical characteristics. I-A tumors were defined by alterations in *ATRX* and in *IDH*; they almost always had ALT and *TP53* mutations, typically had an astrocytic histological component, and were often diagnosed in the fourth decade of life. I-CF tumors were defined by *IDH* mutations and by alterations in either *CIC, FUBP1*, and/or 1p/19q, rarely displayed ALT, typically had an oligodendroglial component and were often diagnosed in the fifth decade of life. This molecular classification system distinguishes the prognosis of gliomas with I-A and I-CF genetic signatures from each other and from the poorly-performing, genetically heterogeneous I-X group (Figure 5).

Our mutational analysis expands on recent reports that *ATRX, CIC*, and *FUBP1* mutations are frequent in specific nervous system tumor types [14, 28-31, 33]. *ATRX* mutations were recently found to define a clinically distinct population of teenaged and young adult neuroblastoma patients, who often had a protracted course with death occurring many years after diagnosis [33]. Our results reveal striking parallels to the *ATRX*-mutated neuroblastoma patients, since *ATRX*-mutated glioma patients are also relatively young adults and demonstrate a protracted but ultimately fatal course (median overall survival 51 months). In contrast to a previous report that *FUBP1* mutations always co-occurred with a *CIC* mutation among a cohort of 18 oligodendrogliomas and 42 oligoastrocytomas reported by Sahm and colleagues [30], we did not observe any significant associations between *CIC* and *FUBP1* mutations among 50 oligodendrogliomas and 40 oligoastrocytomas (Figure 1). However, we did observe a case with *CIC* mutation that occurred in the absence of 1p/19q loss, which is a situation that was observed in one oligodendroglial tumor by Sahm and colleagues [30]. In addition to expanding on previous preliminary reports of the frequency of *ATRX*, *CIC*, and *FUBP1* mutations in selected tumor types, our study for the first time identifies associations between these mutations and age at diagnosis and clinical outcome among glioma patients.

Mutational analysis of *ATRX*, *CIC*, and *FUBP1* refined the prognostic information provided by the known prognostic markers *IDH*, *TP53*, and 1p/19q [1, 2, 11, 32, 34]. This refinement is shown by comparing tumors with isolated *IDH* mutations to patients who had these mutations in the context of a full I-A or I-CF genetic signature (Figure 4). 1p/19q status alone also may not be sufficient to determine whether a tumor contains alterations in *CIC* or *FUBP1*, since *CIC* mutation occurred in the absence of 1p/19q alteration in one of the cases studied. Also, the presence of an oligoastrocytoma with “mixed” I-A and I-CF signatures raises the possibility that a subset of histopathologically mixed tumors are truly heterogeneous in a manner that may not be detected by routine 1p/19q analysis alone. *IDH*-mutated gliomas are a subset of the gliomas with the proneural gene expression signature [6], and *IDH* mutations tightly overlap with the G-CIMP hypermethylator phenotype among gliomas [8, 35]. Therefore, the I-A and I-CF genetic signatures provide a molecular platform to further subclassify the broader proneural and G-CIMP molecular subgroups of gliomas.

In contrast to the I-A and I-CF genetic signatures, which represent homogeneous genetic disease entities, I-X tumors are a genetically heterogeneous group. Based on their association with poor patient survival and advanced patient age, and on their lack of *IDH* or *ATRX* mutations, I-X tumors are similar to primary GBMs [3, 10, 13] and to *IDH* wild-type low-grade gliomas [36, 37]. Patients with these tumors can be further classified into prognostic subgroups on the basis of other mutational, copy number, gene expression, and DNA methylation alterations such as *EGFR* amplification, *PTEN* deletion, gene expression signatures, and other previously-described markers [3-8]. Our genetic classification scheme provides a prognostic tool to identify uncommon I-A and I-CF tumors among the grade IV gliomas and to classify the vast majority of grade II-III gliomas.

Many tumors classified as one glioma subtype by histopathological criteria bore hallmark genetic and clinical features of another tumor type in the present study. 16% (7/39) of oligodendrogliomas and 3% (3/87) of primary GBMs bore the I-A genotype, suggesting that a subset of tumors diagnosed as oligodendrogliomas or primary GBMs are genetically more similar to grade II-III astrocytomas. 11% (15/132) of grade II-III gliomas and GBMs lacked I-A or I-CF gene signatures altogether and had a poor survival (I-X tumors) and are likely genetically more similar to primary GBMs than to other grade II-III gliomas. Moreover, 98% (39/40) of gliomas containing histopathological features of both oligodendrogliomas and astrocytomas could be unambiguously assigned to a genetic signature: 26 were I-A, 9 were I-CF, and 4 were I-X.

Currently, there are no significant differences in the treatment of gliomas based on astrocytic or oligodendrocytic differentiation, or based on any molecular prognostic markers. Treatment decisions are based on WHO tumor grade [2, 38] despite the fact that outcomes vary significantly between different glioma subgroups [1, 7, 25, 27]. With the development of personalized therapies for glioma patients, a refined classification that incorporates genetic signatures may become critical for their management. As sequencing technology declines in cost, genotypic analysis is expected to become increasingly accessible to patients, not only as part of clinical trials, but also as part of standard clinical care. In the interim, these data suggest that new therapeutic trials for patients with gliomas should incorporate genetic signatures, as well as histopathologic signatures, to help to classify patients for eligibility for a specific treatment and to determine their outcomes. Furthermore, the identification in this study of genetic alterations that may cooperate in driving glioma pathogenesis will inform future studies of the molecular mechanism of glioma pathogenesis and the design of targeted therapies.

## METHODS

### Patient Samples

Snap-frozen tumor tissue and matched blood was obtained from patients from The Preston Robert Tisch Brain Tumor Center Biorepository at Duke University Medical Center (n=291), Johns Hopkins Medical Institutes (n=27), and the University of Sao Paulo, Brazil (n=45) with informed consent from the patients under protocols approved by the Institutional Review Boards of the respective instituions. Tissue sections were centrally reviewed by board-certified neuropathologists to ensure that ≥ 95% of the section consisted of tumor cells and to confirm histopathological diagnosis. DNA was extracted from snap-frozen tissue obtained from primary brain tumors or from xenografts that were serially passaged in mice that were derived from primary brain tumors. All samples were obtained in accordance with the Health Insurance Portability and Accountability Act (HIPAA). 9 grade I pilocytic astrocytomas, 15 grade II astrocytomas, 44 grade III astrocytomas, 15 grade IV secondary GBMs, 94 grade IV primary GBMs, 25 grade IV pediatric GBMs, 21 grade II oligodendrogliomas, 29 grade III oligodendrogliomas, 18 grade II oligoastrocytomas, 22 grade III oligoastrocytomas, 65 medulloblastomas, 2 pleomorphic xanthoastrocytoma, and 5 ependymomas were sequenced. Secondary GBM designates a GBM which was resected > 1 year after a prior diagnosis of a lower grade glioma (grade I-III). Pediatric GBM samples were defined if between the ages of 0-20 and adult GBM was classified as ages 21+. One grade IV GBM was not included in mutation frequency analyses as described below. Pilocytic astrocytomas, pleomorphic xanthoastrocytomas, ependymomas, and medulloblastomas were not considered for clinical analyses of grade II-IV gliomas. Median and mean ages reported in Table 1 was based on patients with available clinical information. Patients with survival of 1 month or less, likely due to surgical complications, were excluded from the survival analysis.

### Sequencing

DNA was isolated from frozen tissue and the entire coding sequences of *ATRX*, *CIC*, and *FUBP1* were analyzed by Sanger sequencing [39] for 363 tumors. 1p and 19q copy number was determined by microsatellite marker analysis and mutational status of *TP53*, *IDH1*, and *IDH2* was determined, as described previously [10]. All coding exons of *ATRX*, *CIC*, and *FUBP1* were examined in 363 tumors for this study. *Death domain associated protein* (*DAXX*) was sequenced in all grade II-III astrocytomas and oligodendrogliomas because it is mutated in pancreatic neuroepithelial tumors and pediatric GBMs [14, 31] and because it functions in the same telomere-regulating pathway as *ATRX* [17]. No *DAXX* mutations were found in those gliomas. *ATRX* mutational status (but not *CIC* or *FUBP1* mutational status) for a subset of GBMs, medulloblastomas, and 13 oligodendrogliomas were reported in a previous resequencing study [13]. No grade II-III gliomas aside from those 13 oligodendrogliomas were examined for *ATRX* in that analysis. *CIC* and *FUBP1* mutational status for 7 oligodendrogliomas were reported in a previous whole-exome sequencing study [28]. *CIC* and *FUBP1* status from other oligodendrogliomas, and for non-oligodendroglioma brain tumors reported here were not included in that analysis. Sequencing was performed by PCR amplification followed by Sanger sequencing as described previously [39].

For *IDH1*, *IDH2*, *TP53*, and 1p/19q status, sequencing efforts on an overlapping panel of 445 brain tumors for those alterations [10] were supplemented by analyzing these markers in all remaining tumors for which additional tumor DNA or tissue was available. *IDH1* and *IDH2* status were based on the presence or absence of either R132 or R172 mutations, respectively, from exon 4 sequencing of either gene. *TP53* mutational status, as well as 1p and 19q chromosomal status based on microsatellite markers, were determined as described previously [10]. All mutations were validated with Sanger sequencing in tumors and matched normal controls. One sample pGBM IV-2, harbors a somatic deletion adjacent to the intron exon edge of *ATRX* and is likely to affect functionality; this sample was considered to have an *ATRX* mutation for the purposes of this study. Another sample, GBM-13, had a “hypermutator” phenotype and harbored an extreme number of mutations that were likely to be passenger mutations, as discussed in a previous exomic sequencing analysis [5]. This case was excluded from analyses of mutation frequency, mutation co-occurrence, and patient characteristics.

### Telomere FISH, and Immunolabeling

Telomere length was assessed by FISH in 71 samples with available formalin-fixed, paraffin-embedded tissue, and p53 labeling was performed by immunofluorescence [13, 24, 31, 40, 41]. Loss of *ATRX* nuclear immunolabeling indicates that *ATRX* is inactivated by genetic or epigenetic changes not identifiable by sequencing of *ATRX* exons [13, 42], therefore tumors with *ATRX* mutation and/or loss of ATRX nuclear immunolabeling in tumor cells but not normal cells as assessed by immunohistochemistry [13] were considered to have an ATRX alteration. Fisher's exact test was used to determine whether two aberrations were associated among a group of patients.

Deparaffinized slides were hydrated, steamed for 20 minutes in citrate buffer (catalog# H-3300; Vector Laboratories), dehydrated and hybridized with a Cy3-labeled peptide nucleic acid (PNA) probe complementary to the mammalian telomere repeat sequence ([N-terminus to C-terminus] CCCTAACCCTAACCCTAA). Following post-hybridization washes, the desired primary antibody was applied (anti-ATRX, as described above), followed by application of species-appropriate Alexa 488 fluorescent secondary antibody (Molecular Probes Cat.# A-11034 or A-11001) and nuclear counterstaining with DAPI. Slides were imaged with a Nikon 50i epifluorescence microscope equipped with X-Cite series 120 illuminator (EXFO Photonics Solutions Inc., Ontario, CA) and appropriate fluorescence excitation/emission filters. Grayscale images were captured using Nikon NIS-Elements software and an attached Photometrics CoolsnapEZ digital camera, pseudo-colored and merged.

The FISH and immune-labeled slides were assessed and scored independently by 2 authors (C.H. and A.K.M.). Large, ultra-bright telomere repeat DNA aggregates are unique to ALT-positive cell populations and are significantly larger and brighter than the FISH signals emanating from individual telomeres in the same cell population (for further details, see detailed discussion of these foci as markers for the ALT phenotype in Supplementary Online Material of [13]). In this study, gliomas were classified as ALT-positive if they met the following criteria: (i) the presence of ultra-bright, intra-nuclear foci of telomere FISH signals, with signal intensities and areas for individual foci being significantly larger than any telomeric signals in individual benign cells within the same case and (ii) ≥1% of neoplastic cells displaying ALT-associated telomeric DNA foci. Tumor samples lacking ALT-associated telomeric foci in which at least 5000 cells were assessed were considered ALT-negative. In all cases, areas exhibiting necrosis were excluded from consideration.

### ATRX Immunohistochemistry

Heat-induced antigen retrieval was performed in a steamer using citrate buffer (catalog# H-3300, Vector Laboratories) for 30 minutes. Endogenous peroxidase was blocked (catalog# S2003, Dako) and serial sections were then incubated with primary antibody; anti-ATRX (1:400 dilution; catalog# HPA001906, Sigma-Aldrich, lot R00473) for 1 hour at room temperature. The primary antibodies were detected by 30 minute incubation with HRP-labeled secondary antibody (catalog# PV6119, Leica Microsystems) followed by detection with 3,3′Diaminobenzidine (Sigma-Aldrich), counterstaining with Harris hematoxylin, rehydration and mounting. Only nuclear labeling of either protein was evaluated. The immune-labeled glioma slides were assessed and scored by 3 authors (R.F.W., F.R., and A.K.M.); internal controls included endothelial cells (including within intratumoral vessels), and benign neurons, which demonstrated strong nuclear immunolabeling for ATRX. 29 tumors were analyzed for ATRX nuclear staining, 14 of which were also analyzed by sequence analysis. Tumors were considered to have an ATRX alteration if either a mutation was present when analyzed by sequencing, or if ATRX nuclear expression was lost when tumor tissue was analyzed by immunostaining, as done previously [13]. p53 nuclear protein immunohistochemistry was performed as described previously [41]. Of the 93 tumors that were considered to have ATRX alterations, eight tumors without a detectable ATRX mutation demonstrated loss of ATRX nuclear staining in tumor cells but not normal cells.

### Survival Analysis

Survival was analyzed for adult patients (>20 years old at diagnosis) with grade II-IV gliomas with available survival and age information. 156 patients from Duke (Cohort A) and 43 patients from Brazil (Cohort B) met these criteria. A Mantel-Cox log-rank test was used to determine whether survival was significantly different between two or more groups of patients. A two-tailed Student's t-test and single factor ANOVAs were used to determine whether mean age was significantly different between two or three groups of patients, respectively.

## Supplementary Tables and Figures


