# Supplementary material for: Frequent ATRX, CIC, FUBP1 and IDH1 mutations refine the classification of malignant gliomas
Source: Oncotarget. 2012 Aug 3;3(7):709–22. doi: 10.18632/oncotarget.588 (PMC3443254; doi:10.18632/oncotarget.588)
Supplement: Supplementary file 1 [file oncotarget-03-709-s001.pdf]

Frequent *ATRX*, *CIC*, and *FUBP1* mutations refine the classification of malignant gliomas – Jiao et al

Supplementary Table 1. *ATRX*, *CIC*, and *FUBP1* mutation details.

Sample ID	Gene	Nucleotide (genomic)*	Amino acid$ (protein)	Mutation type	Mutation Location (Exon)	Domain
GBM IV-3	*ATRX*	g.chrX:76838758_76838759AT>C(hom)	frameshift	indel	5	
GBM IV-5	*ATRX*	g.chrX:76831065delG(hom)	frameshift	indel	7	ADD
A III-22	*ATRX*	g.chrX:76831012delT	frameshift	indel	7	ADD
OA II-1	*ATRX*	g.chrX:76831012delT	frameshift	indel	7	ADD
GBM IV-6	*ATRX*	g.chrX:76831000A>C	p.Y187X	nonsense	7	ADD
OA III-9	*ATRX*	g.chrX:76827136het_delT	frameshift	indel	8	ADD
O II-10	*ATRX*	g.chrX:76827087C>A	p.R221M	missense	8	ADD
A II-3	*ATRX*	g.chrX:76826742G>C(hom)	frameshift	splice site	in9	ADD
A III-1	*ATRX*	g.chrX:76826646A>T	p.L253X	nonsense	9	ADD
GBM IV-9	*ATRX*	g.chrX:76826615C>T(hom)	p.W263X	nonsense	9	ADD
O III-1	*ATRX*	g.chrX:76826597_76826603delGTGACAA(hom)	frameshift	indel	9	ADD
A III-14	*ATRX*	g.chrX:76826584_76826585insA(hom)	frameshift	indel	9	ADD
A II-8	*ATRX*	g.chrX:76826530G>A	p.Q292X	nonsense	9	
A III-21	*ATRX*	g.chrX:76826336_76826337insT	frameshift	indel	9	
OA III-14	*ATRX*	g.chrX:76826330delT(hom)	frameshift	indel	9	
OA II-4	*ATRX*	g.chrX:76826328_76826329insT	frameshift	indel	9	
A II-2	*ATRX*	g.chrX:76826328_76826329insT	frameshift	indel	9	
A III-12	*ATRX*	g.chrX:76826327_76826330delCAGT	frameshift	indel	9	
GBM IV-1	*ATRX*	g.chrX:76826327_76826330delCAGT	frameshift	indel	9	
GBM IV-2	*ATRX*	g.chrX:76826245delT	frameshift	indel	9	
A III-6	*ATRX*	g.chrX:76826130_76826131delTT	frameshift	indel	9	
OA III-11	*ATRX*	g.chrX:76825972delC(hom)	frameshift	indel	9	
A II-4	*ATRX*	g.chrX:76825807C>A(hom)	p.E533X	nonsense	9	
A III-23	*ATRX*	g.chrX:76825807C>A(hom)	p.E533X	nonsense	9	
OA II-6	*ATRX*	g.chrX:76825753C>A	p.G551X	nonsense	9	
A II-7	*ATRX*	g.chrX:76825743_76825744delTG	frameshift	indel	9	
GBM IV-10	*ATRX*	g.chrX:76825743_76825744delTG	frameshift	indel	9	
A II-9	*ATRX*	g.chrX:76825412_76825413insAAGGGTGTAGTCATTCTTCATCTGAATTAGATGTT ACAGGGTTAGTTTCTGTCGGTCGCCTC	frameshift	indel	9	
O II-11	*ATRX*	g.chrX:76825234_76825235delTC	frameshift	indel	9	
A III-27	*ATRX*	g.chrX:76825229_76825230delCA	frameshift	indel	9	
GBM IV-4	*ATRX*	g.chrX:76825188_76825194delTTGAGGA	frameshift	indel	9	
A III-3	*ATRX*	g.chrX:76825164_76825165delCT(hom)	frameshift	indel	9	
OA II-9	*ATRX*	g.chrX:76825118_76825163delCTTATGGTTTGTATGAATTTCATTAATATCAGTAT CTGAAGAAGAA(hom)	frameshift	indel	9	
A III-20	*ATRX*	g.chrX:76825069_76825075delTTTTTCC(hom)	frameshift	indel	9	
A III-25	*ATRX*	g.chrX:76825063G>A(hom)	p.R781X	nonsense	9	
A III-15	*ATRX*	g.chrX:76825041G>C(hom)	p.S788X	nonsense	9	
A II-6	*ATRX*	g.chrX:76825014delG	frameshift	indel	9	
A II-5	*ATRX*	g.chrX:76824982G>A	p.R808X	nonsense	9	
A III-24	*ATRX*	g.chrX:76824982G>A	p.R808X	nonsense	9	
OA II-7	*ATRX*	g.chrX:76824777delG(hom)	frameshift	indel	9	
O III-15	*ATRX*	g.chrX:76824745_76824748delTCTC(hom)	frameshift	indel	9	
A III-2	*ATRX*	g.chrX:76824595T>A(hom)	p.R937X	nonsense	9	
A III-16	*ATRX*	g.chrX:76824424_76824425insT	frameshift	indel	9	
A III-10	*ATRX*	g.chrX:76824273_76824277delTTTTC(hom)	frameshift	indel	9	
A III-28	*ATRX*	g.chrX:76824267_76824271delCTTTT	frameshift	indel	9	

OA III-5	*ATRX*	g.chrX:76824267_76824271delCTTTT	frameshift	indel	9	
OA III-12	*ATRX*	g.chrX:76824258delA(hom)	frameshift	indel	9	
A III-8	*ATRX*	g.chrX:76824256_76824257delTT(hom)	frameshift	indel	9	
O II-7	*ATRX*	g.chrX:76824089delT	frameshift	indel	9	
A III-13	*ATRX*	g.chrX:76824059A>C	p.Y1115X	nonsense	9	
OA III-15	*ATRX*	g.chrX:76823706_76823707het_delAA(hom)	frameshift	indel	9	
A III-17	*ATRX*	g.chrX:76823670G>C	p.S1245X	nonsense	9	
GBM IV-8	*ATRX*	g.chrX:76806828_76806829insT	frameshift	indel	11	
O II-1	*ATRX*	g.chrX:76806828_76806829insT	frameshift	indel	11	
OA III-3	*ATRX*	g.chrX:76805654_76805655insA	frameshift	indel	12	
OA II-10	*ATRX*	g.chrX:76805622delC	frameshift	indel	12	
OA III-1	*ATRX*	g.chrX:76805531_76805534delTCTT	frameshift	indel	12	
pedGBM IV-1	*ATRX*	g.chrX:76798774_76798775delAG(hom)	frameshift	indel	13	
A III-18	*ATRX*	g.chrX:76798761delA	frameshift	indel	13	
pedGBM IV-4	*ATRX*	g.chrX:76798738_76798741delACTA	frameshift	indel	13	
OA III-10	*ATRX*	g.chrX:76796285G>A	p.R1426X	nonsense	14	
A II-11	*ATRX*	g.chrX:76796254delC	frameshift	indel	14	
GBM IV-14	*ATRX*	g.chrX:76794307G>A	p.R1504X	nonsense	15	
O II-3	*ATRX*	g.chrX:76794277G>A(hom)	p.R1514X	nonsense	15	
M IV-2	*ATRX*	g.chrX:76778161_76778162insA	frameshift	indel	16	
OA II-8	*ATRX*	g.chrX:76776798_76776801delTTTC(hom)	frameshift	indel	17	SNF2 Helicase
OA II-5	*ATRX*	g.chrX:76776798_76776801delTTTC	frameshift	indel	17	SNF2 Helicase
OA III-7	*ATRX*	g.chrX:76775702_76775710delTAAGTTACC(hom)	frameshift	indel	18	SNF2 Helicase
A III-5	*ATRX*	g.chrX:76775378_76775381delTTTC	frameshift	indel	19	SNF2 Helicase
OA III-13	*ATRX*	g.chrX:76762576G>A	p.R1739X	nonsense	20	SNF2 Helicase
A III-9	*ATRX*	g.chrX:76762551G>T(hom)	p.T1747K	missense	20	SNF2 Helicase
A II-10	*ATRX*	g.chrX:76761054_76761077delTCCTTAATGGATCCAAGTAAATTT	frameshift	indel	21	SNF2 Helicase
GBM IV-12	*ATRX*	g.chrX:76760970C>T	p.R1803H	missense	21	SNF2 Helicase
A III-7	*ATRX*	g.chrX:76760968C>G	p.A1804P	missense	21	SNF2 Helicase
A III-26	*ATRX*	g.chrX:76741689delT(hom)	frameshift	indel	25	
pedGBM IV-3	*ATRX*	g.chrX:76741670_76741673delCTAT(hom)	frameshift	indel	25	
pedGBM IV-2	*ATRX*	g.chrX:76741530_76741533delACTC	del exon and intron	indel	in25	
A III-4	*ATRX*	g.chrX:76700961_76700964delAAAT(hom)	frameshift	indel	29	C-Terminal Helicase
GBM IV-16	*ATRX*	g.chrX:76700843G>A(hom)	p.R2153C	missense	29	C-Terminal Helicase
GBM IV-11	*ATRX*	g.chrX:76700843G>A(hom)	p.R2153C	missense	29	C-Terminal Helicase
OA III-8	*ATRX*	g.chrX:76699762T>C	p.E2172G	missense	30	C-Terminal Helicase
A III-19	*ATRX*	g.chrX:76699712C>G	p.V2189L	missense	30	C-Terminal Helicase
OA III-6	*ATRX*	g.chrX:76665452_76665465delTTTGTGGTCCAAAA	frameshift	indel	31	
A II-1	*ATRX*	g.chrX:76665420_76665423delTTTC;	frameshift	indel	31	
A III-11	*ATRX*	g.chrX:76665420_76665423delTTTC	frameshift	indel	31	
GBM IV-13	*ATRX*	g.chrX:76665385C>A	frameshift	splice site	in31	
O III-2	*CIC*	g.chr19:47482765_47482766delTG(hom)	frameshift	indel	2	
O III-7	*CIC*	g.chr19:47482849_47482850insC	frameshift	indel	2	Pro-rich
O III-3	*CIC*	g.chr19:47483213_47483214delGA(hom)	frameshift	indel	3	
O III-5	*CIC*	g.chr19:47483555C>T(hom)	p.R201W	missense	5	HMG Box
O III-17	*CIC*	g.chr19:47483555C>T(hom)	p.R201W	missense	5	HMG Box
O II-6	*CIC*	g.chr19:47483558C>T(hom)	p.R202W	missense	5	HMG Box
O III-18	*CIC*	g.chr19:47483592G>C(hom)	p.R213P	missense	5	HMG Box
O III-19	*CIC*	g.chr19:47483598G>A	p.R215Q	missense	5	HMG Box
O III-10	*CIC*	g.chr19:47483630G>T(hom)	p.D226Y	missense	5	HMG Box
O II-5	*CIC*	g.chr19:47483658G>A	p.G235D	missense	5	HMG Box
M IV-3	*CIC*	g.chr19:47485356C>T	p.R440C	missense	8	
O III-14	*CIC*	g.chr19:47485924_47485925insG	frameshift	indel	9	
GBM IV-7	*CIC*	g.chr19:47486450C>T(hom)	p.Q564X	nonsense	10	Pro-rich
O III-16	*CIC*	g.chr19:47486575_47486576delTG(hom)	frameshift	indel	10	Pro-rich

O III-4	*CIC*	g.chr19:47486736_47486752delCCCCAGCAGCTCCCCTG(hom)	frameshift	indel	10	Pro-rich
O II-8	*CIC*	g.chr19:47487082_47487083insCCCCCTG(hom)	frameshift	indel	10	Pro-rich
O II-15	*CIC*	g.chr19:47487082_47487083insCCCCCTG(hom)	frameshift	indel	10	Pro-rich
OA III-4	*CIC*	g.chr19:47487095C>T	p.Q779X	nonsense	10	Pro-rich
O II-13	*CIC*	g.chr19:47487319_47487323delCAGCG(hom)	frameshift	indel	10	Pro-rich
O III-21	*CIC*	g.chr19:47487549G>A(hom)	frameshift	splice site	in10	Pro-rich
O II-2	*CIC*	g.chr19:47487580_47487581insGC	frameshift	indel	11	Pro-rich
O II-4	*CIC*	g.chr19:47488077G>A	frameshift	splice site	in12	Pro-rich
OA II-2	*CIC*	g.chr19:47489118C>T	p.R1214X	nonsense	15	Pro-rich
OA III-2	*CIC*	g.chr19:47489221delC	frameshift	indel	15	Pro-rich
O III-6	*CIC*	g.chr19:47489795delC(hom)	frameshift	indel	16	
O III-11	*CIC*	g.chr19:47489819_47489820delCT(hom)	frameshift	indel	16	
M IV-1	*CIC*	g.chr19:47490170G>C	p.D1401H	missense	18	
O III-13	*CIC*	g.chr19:47490688G>T(hom)	p.V1474F	missense	19	
O II-12	*CIC*	g.chr19:47490903_47490905delAGA	p.1517delK	indel	20	
GBM IV-7	*FUBP1*	g.chr1:78208234_78208235insATAACCA	frameshift	indel	2	
O II-14	*FUBP1*	g.chr1:78208200_78208201insC	frameshift	indel	2	
O II-16	*FUBP1*	g.chr1:78206473delG	frameshift	indel	3	
O III-9	*FUBP1*	g.chr1:78206436_78206439delCAGT(hom)	frameshift	indel	3	
GBM IV-15	*FUBP1*	g.chr1:78205024C>A	frameshift	splice site	7	
O III-12	*FUBP1*	g.chr1:78202995_78202996insCT	frameshift	indel	in10	
GBM IV-13	*FUBP1*	g.chr1:78202625G>A	p.P281L	missense	11	KH3
O III-20	*FUBP1*	g.chr1:78201156C>A	p.E411X	nonsense	14	KH4
O III-14	*FUBP1*	g.chr1:78201054C>A	p.E445X	nonsense	14	
O III-17	*FUBP1*	g.chr1:78198726delG(hom)	frameshift	indel	15	
O III-5	*FUBP1*	g.chr1:78198726delG(hom)	frameshift	indel	15	
OA III-2	*FUBP1*	g.chr1:78198455A>G	frameshift	splice site	16	
O III-3	*FUBP1*	g.chr1:78194962CT>C	p.AT530A	indel	17	
O III-8	*FUBP1*	g.chr1:78194940_78194941insT(hom)	frameshift	indel	17	
OA II-3	*FUBP1*	g.chr1:78194860_78194861insT	frameshift	indel	17	
O II-9	*FUBP1*	g.chr1:78187532G>A	p.Q608X	nonsense	19	
O III-2	*FUBP1*	g.chr1:78187498_78187499delTA(hom)	frameshift	indel	19	

*Coordinates refer to human reference genome hg18 release (NCBI 36.1, March 2006).

$ Single-letter abbreviations for the amino acid residues are as follows: A, Ala; C, Cys; D, Asp; E, Glu; F, Phe; G, Gly; H, His; K, Lys; L, Leu; M, Met; P, Pro; Q, Gln; R, Arg; S, Ser; T, Thr; V, Val; W, Trp; and X, STOP.

^Sample ID indicates tumor abbreviation followed by WHO grade. Abbreviations are as follows: AII, astrocytoma grade II; AIII, astrocytoma grade III; GBM IV, GBM grade IV; pedGBM, pediatric GBM grade IV; OII, oligodendroglioma grade II; OIII, oligodendroglioma grade III; OAII, oligoastrocytoma grade II; OAIII, oligoastrocytoma grade III; M IV, Medulloblastoma grade IV.

Supplementary Table 2. Clinical characteristics from glioma patients in two cohorts.

	Cohort A*	Cohort B*
Total No. patients	156	43
No. grade II (%)	27 (17)	4 (9)
No. grade III (%)	61 (39)	8 (19)
No. grade IV (%)	68 (44)	31 (72)
No. oligodendroglial (%)	7 (5)	0 (0)
No. mixed histology (%)	44 (28)	0 (0)
No. astrocytic (%)	105 (67)	43 (100)
No. I-A (%)	47 (30)	12 (28)
Mean age at diagnosis ± S.D., years	35 ± 8	30 ± 5
Median survival, months	59	43
No. I-X (%)	70 (45)	30 (70)
Mean age at diagnosis ± S.D., years	56 ± 14	50 ± 14
Median survival, months	17	11
No. I-CF (%)	39 (25)	1 (2)
Mean age at diagnosis ± S.D., years	43 ± 13	53
Median survival, months	94	N.R.

*Cohort A was from Duke University Medical Center, United States. Cohort B was from the University of Sao Paulo, Brazil.

Supplementary Table 3. Characteristics of mixed histology tumors analyzed in this study.

Sample ID	Diagnosis	Age	Sex	ATRX	ATRX IHC	ALT Staining	IDH1	IDH2	TP53	CIC	FUBP1	1p and 19q LOH	I-A, I-CF or I-X
1	OAII	38	F	MUT	NA	NA	MUT	no	MUT	no	no	no	I-A
2	OAII	80	M	no	Positive	Negative	MUT	no	no	no	no	no	I-X
3	OAII	45	M	no	NA	NA	MUT	no	no	no	no	Yes	I-CF
4	OAII	52	F	no	Positive	Negative	MUT	no	no	no	no	Yes	I-CF
5	OAII	21	F	no	Negative	Positive	MUT	no	MUT	no	no	no	I-A
6	OAII	52	F	MUT	NA	NA	MUT	no	MUT	no	no	no	I-A
7	OAII	38	M	MUT	Negative	Positive	MUT	no	MUT	no	no	no	I-A
8	OAII	51	M	no	Positive	Negative	MUT	no	no	no	no	no	I-X
9	OAII	35	F	MUT	Negative	Positive	MUT	no	MUT	no	no	no	I-A
10	OAII	61	M	no	NA	NA	MUT	no	MUT	no	no	no	I-X
11	OAII	52	M	MUT	Negative	Positive	MUT	no	MUT	no	no	no	I-A
12	OAII	25	F	no	Positive	Negative	MUT	no	no	MUT	no	no	I-CF
13	OAII	30	M	MUT	Negative	Positive	MUT	no	MUT	no	no	no	I-A
14	OAII	32	M	no	Negative	Positive	MUT	no	MUT	no	no	no	I-A
15	OAII	32	M	no	Positive	Negative	no	MUT	no	no	no	Yes	I-CF
16	OAII	34	M	MUT	Negative	Positive	MUT	no	MUT	no	no	no	I-A
17	OAII	39	F	MUT	Negative	Positive	MUT	no	MUT	no	no	no	I-A
18	OAII	46	M	no	NA	NA	MUT	no	no	no	MUT	Yes	I-CF
19	OAIII	65	M	MUT	Negative	Positive	MUT	no	no	no	no	no	I-A
20	OAIII	38	F	MUT	Negative	Positive	MUT	no	MUT	no	no	no	I-A
21	OAIII	44	F	MUT	NA	NA	MUT	no	MUT	no	no	no	I-A
22	OAIII	49	M	no	Positive	Negative	MUT	no	no	no	no	Yes	I-CF
23	OAIII	31	M	MUT	Negative	Positive	MUT	no	MUT	no	no	no	I-A
24	OAIII	34	F	MUT	Negative	Positive	MUT	no	MUT	no	no	no	I-A
25	OAIII	25	M	MUT	Negative	Positive	MUT	no	MUT	no	no	no	I-A
26	OAIII	32	M	MUT	Negative	Positive	MUT	no	MUT	no	no	no	I-A
27	OAIII	32	M	no	Negative	Positive	MUT	no	MUT	no	no	no	I-A
28	OAIII	26	F	MUT	Negative	Positive	MUT	no	MUT	no	no	no	I-A
29	OAIII	49	F	MUT	NA	NA	MUT	no	MUT	no	no	no	I-A
30	OAIII	30	M	MUT	Negative	Positive	MUT	no	MUT	no	no	no	I-A
31	OAIII	55	M	MUT	NA	NA	MUT	no	MUT	no	no	no	I-A
32	OAIII	48	M	no	Positive	Negative	MUT	no	no	MUT	no	Yes	I-CF
33	OAIII	22	F	no	Negative	Positive	MUT	MUT	MUT	no	no	no	I-A
34	OAIII	44	F	no	NA	NA	no	no	no	no	no	no	I-X
35	OAIII	2	F	MUT	NA	Positive	MUT	no	MUT	no	no	no	I-A
36	OAIII	71	M	no	NA	NA	MUT	no	no	no	no	Yes	I-CF
37	OAIII	42	M	no	Negative	Positive	MUT	no	MUT	no	no	Yes	Mixed I-A and I-CF
38	OAIII	30	M	no	Negative	Positive	MUT	no	MUT	no	no	no	I-A
39	OAIII	45	F	no	NA	NA	MUT	no	no	MUT	MUT	Yes	I-CF
40	OAIII	35	F	MUT	NA	Positive	MUT	no	MUT	no	no	no	I-A

*OAII, grade II oligoastrocytoma; OAIII, grade III oligoastrocytoma. Age is age at diagnosis.

Supplementary Figure 1. Co-occurrence of *IDH, ATRX, CIC,* and *FUBP1* mutations in 363 brain tumors.

All CNS tumors

	IDH	ATRX	TP53	CIC	FUBP1
**IDH (n=156)**	-	57	62	17	10
**ATRX (n=94)**	95	-	90	1	0
**TP53 (n=126)**	76	67	-	0	0
**CIC (n=29)**	93	3	0	-	24
**FUBP1 (n=16)**	100	0	0	44	-

All adult gliomas

	IDH	ATRX	TP53	CIC	FUBP1
**IDH (n=153)**	-	58	61	18	10
**ATRX (n=88)**	99	-	93	1	0
**TP53 (n=113)**	82	73	-	0	0
**CIC (n=27)**	100	4	0	-	26
**FUBP1 (n=16)**	100	0	0	44	-

Astrocytic Tumors

	IDH	ATRX	TP53	CIC	FUBP1
**IDH (n=68)**	-	78	87	1	3
**ATRX (n=54)**	98	-	93	0	0
**TP53 (n=78)**	78	65	-	0	0
**CIC (n=1)**	100	0	0	-	100
**FUBP1 (n=2)**	100	0	0	50	-

Oligodendroglial tumors

	IDH	ATRX	TP53	CIC	FUBP1
**IDH (n=46)**	-	15	15	50	26
**ATRX (n=7)**	100	-	100	0	0
**TP53 (n=7)**	100	100	-	0	0
**CIC (n=23)**	100	0	0	-	22
**FUBP1 (n=12)**	100	0	0	42	-

Oligoastrocytic (Mixed) tumors

	IDH	ATRX	TP53	CIC	FUBP1
**IDH (n=39)**	-	69	69	8	5
**ATRX (n=27)**	100	-	96	0	0
**TP53 (n=27)**	100	96	-	0	0
**CIC (n=3)**	100	0	0	-	33
**FUBP1 (n=2)**	100	0	0	50	-

IDH Mutant Gliomas

	Oligo.	Mixed Histology	Astrocytic
**ATRX**	15	69	78
**TP53**	15	69	87
**CIC**	50	8	1
**FUBP1**	26	5	3

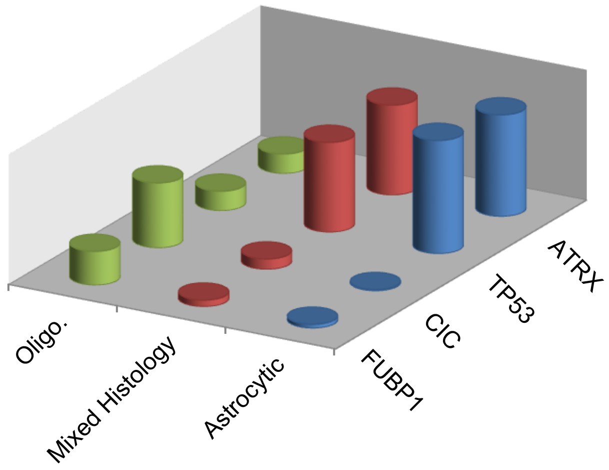

Groups include all CNS tumors (n=363), adult grade II-IV gliomas (n=257), oligodendroglial tumors (n=50), astrocytic tumors (n=167), mixed histology tumors (oligoastrocytomas, n=40), and all IDH-mutated adult gliomas (n=153). The graph displays mutation frequencies of in IDH-mutated astrocytic, oligodendroglial and mixed histology tumors based on histopathological diagnosis (n=153). Astrocytic tumors were defined in this figure as grade II astrocytomas (AII), grade III astrocytomas (AIII), secondary GBMs (2° GBM), and primary GBMs (1° GBM). Percentage of co-occurance is shown.

Supplementary Figure 2. Survival of patients with I-A and I-CF genetic signatures among different glioma subgroups.

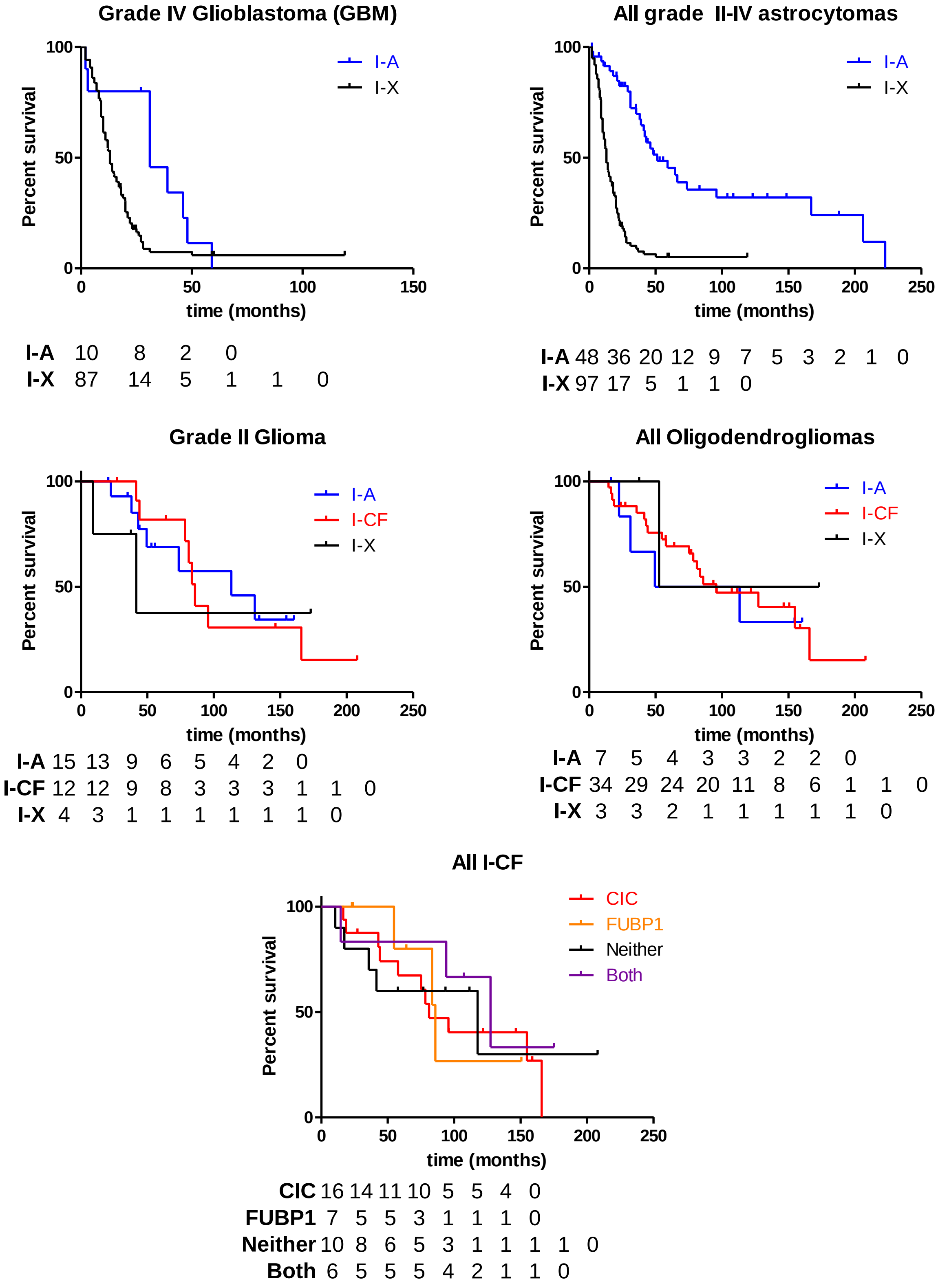

Kaplan-Meier estimates of overall survival are shown for subgroups of 199 patients with grade II-IV gliomas, with the number of patients at risk at each time shown below each respective plot. Survival was significantly different between grade IV glioblastoma patients (n=97) with I-A and I-X signatures (P=0.02, log-rank test). There was a significant difference in survival between astrocytoma patients (grade II-IV, n=145) whose tumors had I-A or I-X signatures (P<0.001, log-rank test). A significant difference in survival between patients with I-A, I-CF, and I-X tumors was not observed among all grade II glioma patients (n=31, P=0.79, log-rank test) or among oligodendroglioma patients (grade II-III, n=44; P=0.76, log-rank test). A significant difference in survival between patients with FUBP1 mutation, CIC mutation, both mutations, or neither mutation among all grade II-IV glioma patients with the I-CF signature (n=39) was not observed (P=0.43, log-rank test).
